# Enthusiasm and structural frustration? Insights from an exploratory cross-sectional two-cohort study into career decision-making and retention factors in German neurology

**DOI:** 10.1186/s12909-026-10515-0

**Published:** 2026-10-05

**Authors:** Georg Prokop, Anne-Sophie Biesalski, Mareike Krause, Pia Stine Rebmann, Johannes Heinrich Alexander Piel, Friederike Schmidt-Graf

**Affiliations:** 1 Department of Neurology, Technical University of Munich, Ismaninger Str. 22, 81675 Munich, Bavaria Germany; 2 Department of Neurology, Hospital Bogenhausen, München Klinik, Englschalkinger Str. 77, 81925 Munich, Bavaria Germany; 3 Department of Neurology, University Hospital Knappschaftsklinik Bochum, Ruhr-University Bochum, In der Schornau 23-25, 44892 Bochum, Germany; 4 Department of Neurology, University Hospital Schleswig-Holstein, Campus Kiel, Arnold-Heller-Str. 3, 24105 Kiel, Germany; 5 Department of Neurology, University Medical Center, University of Rostock, Gehlsheimer Str. 20, 18147 Rostock, Germany

**Keywords:** Neurology, Residency, Workplace conditions, Survey, Career decision

## Abstract

Germany faces critical physician shortages in Neurology. This exploratory study examined factors influencing career decision-making across the Neurology training pipeline in a university setting to inform workforce development strategies. We conducted a cross-sectional two cohort study employing a mixed-methods approach surveying medical students (n = 79) and Neurology residents (n = 56) at German University hospitals via structured questionnaires and open-ended questions. The response rates were 44 % (medical students) and 32 % (residents). The important factors influencing career and medical specialization choices were the specialty itself, good teaching experiences and workplace conditions. Students showed high career motivation. Residents maintained high levels of engagement and specialty loyalty while reporting severe structural deficits in their everyday worklife and residency program. Evidence-based interventions could address these deficits.

**Supplementary Information:**

The online version contains supplementary material available at https://doi.org/10.1186/s12909-026-10515-0.

## Background

Many contemporary health care systems, particularly the German health care system, face severe challenges due to demographic changes. Approximately 25 % of practicing physicians are over the age of 60 and are approaching retirement, while medical school graduation rates remain insufficient for their replacement [1]. This shortage is compounded by approximately 37 % of residents considering changing profession due to workplace conditions [2], potentially creating severe physician shortages.

These challenges coincide with the increase in neurological disease burden driven by the ageing population [3]. In the European Union over 300 million neurological diseases were documented among its 504 million citizens in 2017 [3]. This mismatch of increased demand and (potential) shortage of skilled workers raises the question of how to attract and retain physicians in the field of Neurology.

Medical specialty choice involves complex interactions between individual, institutional, and societal factors. Research has identified work-life balance, intellectual challenges, and clinical and training experiences as key influences on career decisions [4–6]. Younger physicians increasingly prioritize collaborative work environments and structured training [7]. However, these expectations are often unmet in German residency programs [2, 8], creating a critical gap between need and reality.

Despite extensive research on medical career choice, significant gaps remain in our understanding of specialty-specific factors influencing career decisions in Neurology. Most studies examine only a single time point in the decision-making process. Understanding these dynamics might be helpful for developing targeted interventions. This exploratory study examines factors influencing career decision-making and retention at two critical stages of the Neurology career pipeline at university hospitals in Germany. We employed a two cohort cross-sectional design to capture perspectives from medical students and Neurology residents at university hospitals. On this basis, we suggest strategies for future personnel development.

## Methods

### Study design, recruitment and participant characteristics

This cross-sectional, exploratory, two cohort study employed a sequential career-stage approach to examine factors influencing career decision-making in Neurology in a university setting. It was conducted in Germany between May and June 2025. Two distinct cohorts were selected to represent critical decision points in the neurological career pipeline: medical students and neurological residents. A total of 180 medical students and 174 neurological residents of four different university hospitals in Germany were invited to participate in the study, resulting in response rates of 44 % (79/180) for medical students and 32 % (56/174) for neurological residents”.

Table 1 displays the age and sex distribution of the cohorts.

**Table 1:** Demographics of study participants. All neurological residents were recruited at university hospitals

	Medical students	Neurological residents
*participants (response rate)*	79 (43,9 %)	56 (32,2 %)
*female*	53 (67,1 %)	37 (66,1 %)
*male*	23 (29,1 %)	19 (33,9 %)
*non-binary*	3 (3,8 %)	0 (0 %)
*median age (range)*	24 (21 - 45)	30 (26 - 41)

All medical students enrolled in the Neurology lecture at the TUM School of Medicine and Health during summer term of 2025 were contacted via e-mail and asked to participate in the study. The neurology lecture is primarily offered to fourth-year medical students. All neurological residents of four different university hospitals in Germany (TUM University Hospital, University Hospital Schleswig-Holstein, University Medical Centre Rostock, Ruhr University Katholisches Klinikum Bochum) were asked to participate in this survey. They were also contacted via e-mail.

Each cohort had 30 days to complete the survey. Participation was completely voluntary. All participants were informed on the study and publication aims. No reimbursement was offered. Study participation was viewed as consent.

### Questionnaire

The survey consisted of 11 questions for medical students and 36 questions for residents. It was conducted in German and can be viewed in the supplementary information 1. The questionnaire for medical students focused on factors influencing career choice, as they are typically in an active phase of career exploration. In contrast, the questionnaire for residents lay an emphasize on workplace and resident conditions to reflect the experiences within the labor market. Thus, we ensured a high relevance of the questionnaire to the individual context of the cohort.

The questionnaires were developed iteratively by FSG together with all authors. The development team comprised clinicians, medical educators, graduates and students of medical education as well as representatives of Young Neurology (youth society of the German Neurological Association). Items were based on established constructs (e.g. work-life balance, role models, teaching quality and workplace conditions) identified in existing literature on medical career choice and residency conditions to support content validity. Each team member independently reviewed all items for clarity, relevance and completeness, and proposed revisions. This process represents an multidisciplinary expert-based approach to content validity and is supported by its focus on established constructs. The interdisciplinary team ensured that items reflected both theoretical constructs and practical relevance to the target population. No formal psychometric testing, including assessment of reliability or construct validity, was performed owing its exploratory nature. Each questionnaire contained demographic questions, followed by questions answered via a 5-point Likert scale and open-ended questions for qualitative analysis. The questionnaire can be viewed in the supplementary material. It was initially designed as part of a master’s thesis at Hochschule Wismar.

### Data collection and analysis

This study employed a mixed-methods approach, combining quantitative analysis of Likert-scale responses with qualitative content analysis of open-ended questions. Quantitative and qualitative data were analyzed separately before being integrated in the interpretation of findings. The data were collected anonymously via an online-survey tool (survio.com). IP-addresses were not tracked, allowing in principal multiple entries by the same device. This ensured that all contacted residents could participate despite commonly sharing workstations. Open-ended survey responses were analyzed using a qualitative content analysis approach. GP conducted the initial analysis by reviewing each response for its core statements, which were then assigned to thematic categories. These categories were developed inductively based on recurring content across the responses, without a pre-defined codebook. FSG independently reviewed the categorization and any disagreements were resolved through discussion until consensus was reached. Given the exploratory nature of the study and the relatively small number of open-ended responses, no claims of data saturation are made. Quantitative data were analyzed descriptively. Absolute frequencies and percentages were calculated. As the questionnaires were deliberately designed to reflect the specific contexts of each cohort, direct quantitative comparisons between cohorts were not performed. Inferential statistical testing was further limited by the exploratory study design and the small sample size. Quantitative data analysis was performed using R (v 4.4.2). The figures were created using packages “likert” and “ggplot2”.

## Results

Two different cohorts were created to gain insight into the different stages of the career decision-making process. The average response rate was 38 %, with rates of 44% for medical students and 32 % for residents. Most residents (80%; 45) reported to work full-time, and only 20% (11) work part-time. 15 (27%) of the participating residents were in the first two years, 16 (29%) were in their third or fourth, 15 (27%) were in the fifth or sixth, 8 (13%) were in their seventh (or higher) year of residency. 3 (5%) had completed their residency. German Neurology residency has a minimum duration of five years.

### Specialty choice and career goals

Among the medical students 72 % (n = 57) would study medicine again and 76 % (n = 60) were looking forward to practicing medicine. Only 1 student (1 %) would not study medicine and 1 student (1 %) will not work as a doctor. 32 % (n = 25) know which specialty to pursue, 56 % (n = 44) have an idea and 13 % (n = 10) do not know which specialty to choose.

The participants were asked for their desired long term career position. The overall answers were mixed and can be viewed in Table 2. Of special note is, that no residents were seeking a positions as chief physician at a hospital, but 48 % (n = 27) were aiming to be a senior physician at either a university or non-university hospital.

**Table 2:** Participants were asked for their desired long term career goals. Some answers were only available to Neurological residents or Medical students. This is indicated by the term “not applicable”

	Medical students	Neurological residents
*Self-employed at practice or medical center*	32 % (n = 25)	18 % (n = 10)
*Employed at non-university hospital*	22 % (n = 17)	not applicable
*Employed at university hospital*	13% (n = 10)	not applicable
*Employed at practice or medical center*	10% (n = 8)	16%(n = 9)
*Indecisive*	22 % (n = 17)	14 % (n = 8)
*Other plans*	3 % (n = 2)	2 % (n = 1)
*Purely scientific work*	not applicable	14 % (n = 8)
*Senior Physician at non-university hospital*	not applicable	16 % (n = 9)
*Senior Physician at university hospital*	not applicable	32 % (n = 18)
*Chief Physician at any hospital*	not applicable	0 % (n = 0)

### Career decision factors

**Fig. 1:**
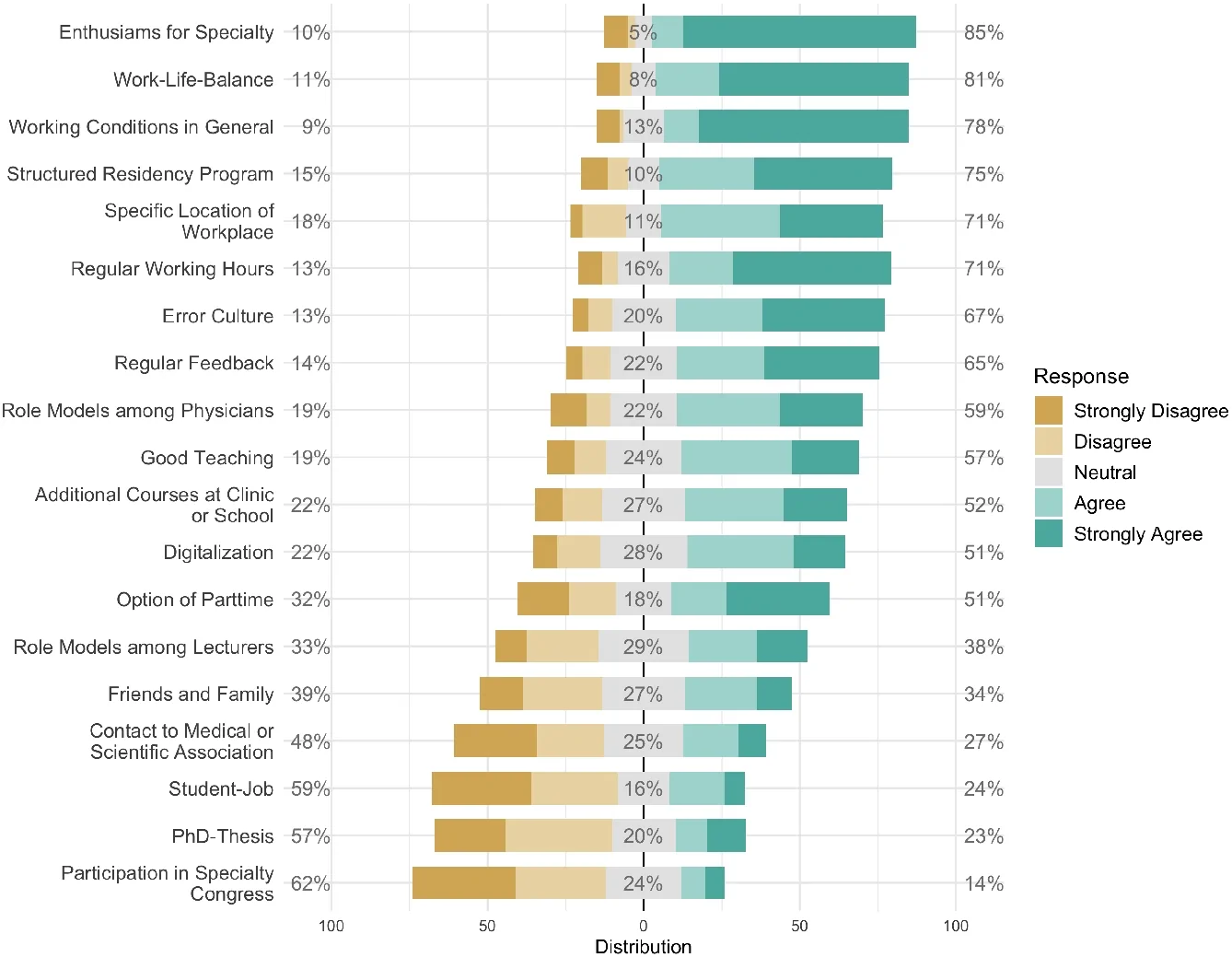
Influence on Career Choice: Medical students were asked to rate the influence these factors have on their career choice on a 5-point Likert scale. The percentage of rejection or agreement is displayed on the left or the right. 79 responses were recorded

**Fig. 2:**
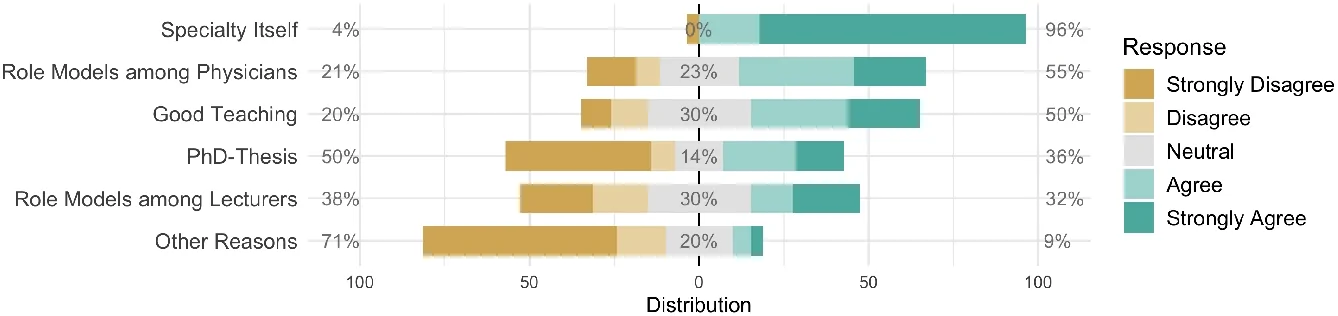
Influence on Career Choice: Neurological residents were asked to rate the influence these factors had on their career choice on a 5-point Likert scale. The percentage of rejection or agreement is displayed on the left or the right. 56 responses were recorded

Medical students and neurological residents were asked to rate the different factors influencing their career choice on a 5-point Likert scale (Figs. 1 and 2). Each bar represents the distribution of responses, with disagreement shown on the left and agreement on the right. The residents were asked to rate retrospectively. Figure 1 displays an representation of the answers provided by medical students; Fig. 2 depicts the answers by residents. Interestingly, the most important factor across the cohorts was the specialty itself (85 % agreement for students and 96 % agreement for residents). Among the top factors for students were also work-life-balance (81 % agreement) and working conditions (78 % agreement) (Fig. 1). Factors related to external influences, such as participation in specialty congresses (14 % agreement), PhD thesis (23 % agrrement) and student jobs (24 % agreement), received the lowest levels of agreement. Residents also endorsed clinician role-models (55 % agreement) and good teaching experiences (50 % agreement) (Fig. 2).

### Qualitative assessment of career decisions

To further illustrate the decision-making process, the students were asked for attracting and deterring factors of a specialty or department and additional comments through open-ended questions. A summary based on the content is presented.

53 students responded for the attracting factors. In summary, students most frequently cited high quality teaching and engaging lectures (30 %, n= 16), as illustrated by comments such as ’Good teaching clearly leads to greater interest in a specialty’. Following as important factors are specific subjects (15 %, n = 8). Furthermore, internships or direct clinical experience with physicians were noted in 11 % (n = 6). Work-life-balance (9 %, n = 5), working conditions (6 %, n = 3) , specialty variety (9 %, n = 5), job satisfaction among doctors (8 %, n = 4), research opportunities (4 %, n = 2), practical courses (4 %, n = 2) and surgical activity (2 %, n = 1) were also listed. 58 students responded for deterring factors. Responses included comments such as ’Impossible working hours, no work-life balance, toxic working environment’ Poor working conditions were the most frequently cited deterrent (40 %, n = 23), followed by negative experiences with physicians or lectures (22 %, n=13) and unfavorable impressions of specific specialties (14 %, n = 8). Other deterring factors included poor teaching quality (10 %, n = 6), negative team dynamics (5 %, n = 3), unfavorable practical experiences (5 %, n = 3), bureaucratic burdens (5 %, n = 3), sexism (3 %, n = 2), monotonous tasks (3 %, n = 2), negative encounters with specific individuals (3 %, n=2). Single mentions include limited specialty diversity, insufficient patient contact, digitization challenges, and economic pressures (2 % each, n = 1). In the additional comments students emphasized among other factors the importance of work-life balance and good working conditions (53 %, n = 9), and the need for respectful treatment of students and staff (11 %, n = 2). Sexism (11 %, n = 2), importance of practical experience (6 %, n = 1), error culture (6 %, n = 1) and a good team spirit (6 %, n = 1) were also mentioned.

In summary, good and practical teaching, positive clinician role models and good workplace conditions are influential factors.

### Residency and workplace conditions

#### Neurological residency

Residents were also asked to rate their neurology residency program. In Germany, the minimum duration of neurology residency is 5 years, although it commonly takes longer. In our cohort, the median time to complete specialist training at the residents’ current institution was 7 years. The median time to complete specialist training at the residents’ current employee was 7 years in our cohort. The specific reported training times were approximately 5 years for 6 % (n = 3), 6 years for 15 % (n = 8), 7 years for 53 % (n = 28), and 8 years for 21 % (n = 11), 10 years for 2 % (n = 1). 9 % (n = 5) did not answer the question. Of all residents 70 % (n = 39) would pursue, 29 % (n = 16) would perhaps pursue and 1 resident (2 %) would not pursue Neurology again as a specialty.

**Fig. 3:**
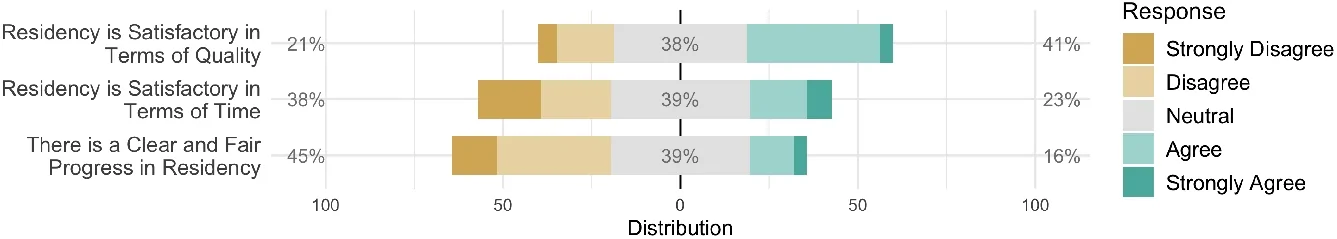
Residency-Program: Neurological residents were asked to rate their residency program on a 5-point Likert-scale. 56 responses were recorded

As shown in Fig. 3 residents were additionally asked to rate their residency program on a 5-point Likert scale. 41 % of the residents reported to be content with the overall quality of their residency program, while 21 % reported dissatisfaction. Satisfaction with the duration of the residency was endorsed by 23 %, with 38 % endorsing dissatisfaction. A clear and fair progress in residency was affirmed by only 16 % of respondents, whereas 45 % disagreed.

Residents were also asked to name important aspects of a good residency program in a free form answer. A total of 46 answers were recorded (82 %). Most (70 %, n = 32) stressed the necessity for early, reliable, and predictable rotations. Additionally, regular (bedside) teaching by attendings and specialists (65 %, n = 30) as well as continuous, organized education (52 %, n = 24) were among the themes most often mentioned. Others involved early and structured training in functional diagnostics (13 %, n = 6), respectful interaction with colleagues and superiors (9 %, n = 4) and regular feedback (7 %, n = 3). Overall, the responses indicate a clear preference for structured, predictable, and professionally sound continuing education with regular supervision and training, supplemented by fair workplace conditions and a supportive, motivating work environment. This is a clash with the reported workplace conditions as shown below.

#### Current working conditions

**Fig. 4:**
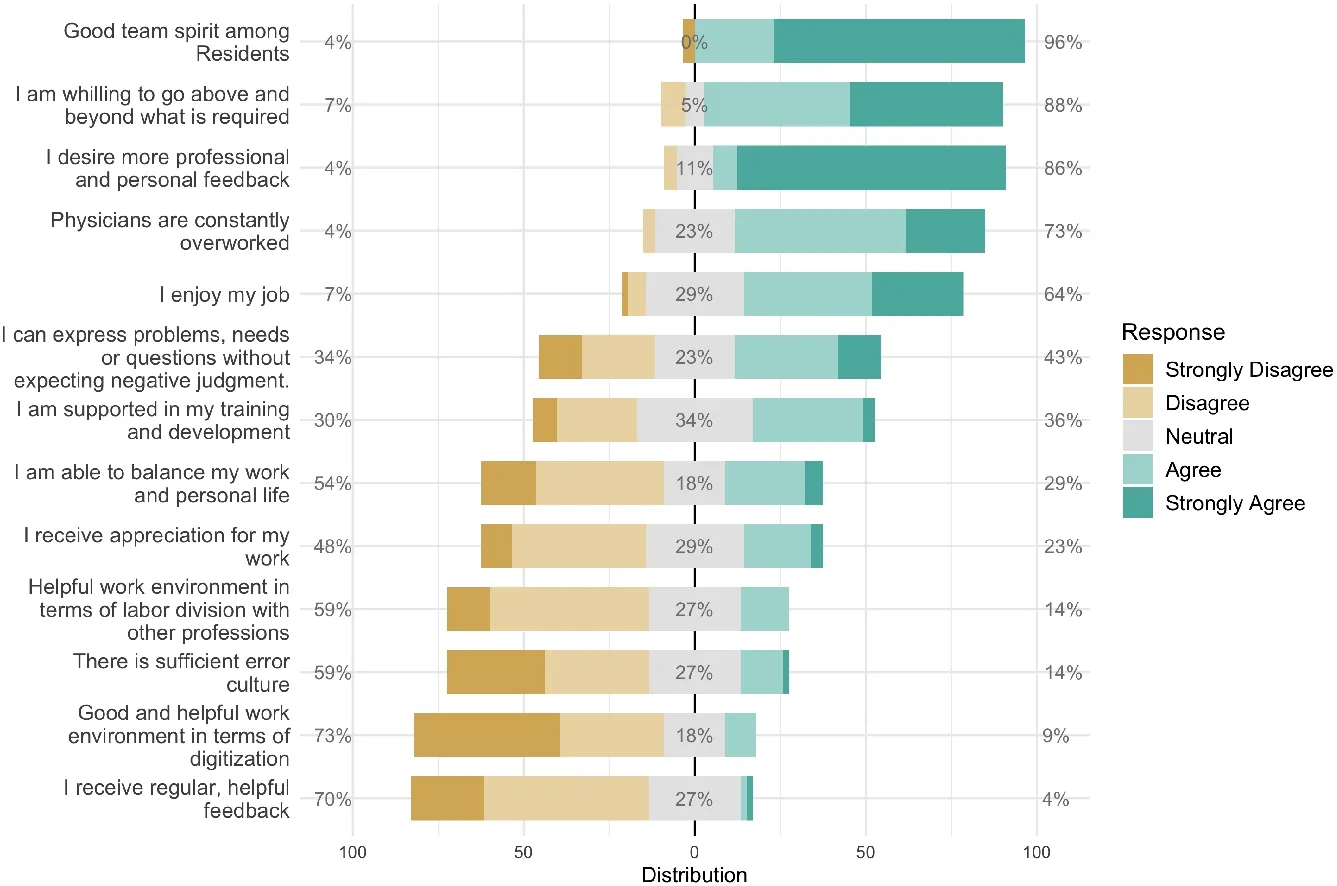
Workplace Conditions: Neurological residents were asked to rate different aspects of the job environment on a 5-point Likert scale. The percentage of rejection or agreement is displayed on the left or the right. 56 responses were recorded

The participants were asked to rate several items related to their current workplace situation. The responses can be seen in Fig. 4. As shown, the above average commitment and good agreement to job satisfaction as well as the good team spirit among residents must be noted. However, the lack of regular feedback, lack of a helpful work environment, lack of a sufficient error culture, struggles to balance work and personal life and the constant overwork of doctors indicate stark structural deficits. When being asked for areas in their workplace that needed improvement in a timely manner, 43 (77 %) participants responded. Residents emphasized concrete needs, for example: ’Fair rotations, close supervision and teaching by senior physicians, fair shift staffing’ and ’More appreciation for staff, fairer rotations and options for leave [...], more teaching, and urgently better IT infrastructure. Currently a lot of duplicate documentation and non-functioning IT.’. As indicated by these comments, the overall answers focus on digitization, appreciation as well as the content and structure of their continuing education. In total numbers digitization and IT-infrastructure were mentioned 16 times (37 %). Additionally the wish for more appreciation (26 %, n = 11), part-time options (21 %, n = 9), more teaching (16 %, n = 7), reliable and predictable rotations during residency program (16 %, n = 7) and fewer non-doctoral/ clerical tasks (16 %, n = 7) were among the answers. Only 41 % (n = 23) would choose their current employer again, 39 % (n = 22) answered this question with “maybe,” and 20 % (n = 11) would not choose their current employer again.

Finally, participants were asked for additional comments. Again, dissatisfaction and frustration surrounding specialist training, the lack of planning security and the lack of appreciation were stressed.

## Discussion

This exploratory, cross-sectional, two-cohort study surveyed medical students and neurological residents at university hospitals. The two cohorts provide insight into the decision-making process at two stages of the career pathway. Our findings support established means to interest students and young physicians in pursuing Neurology as a specialty, while also indicating signs of frustration and structural dissatisfaction despite persistent intrinsic motivation in neurological residents. This study shows an association between structural deficits in workplace conditions and training environments and career dissatisfaction, which in turn might create critical vulnerabilities in workforce development. The proposed measures should be interpreted in light of the exploratory nature of this study as suggestions informed by literature rather than mere direct consequences of the presented data.

### Choosing a specialty and boosting interest

Among the most important factors influencing career choice in this study were the specialty itself, positive training experiences, workplace culture and clinician role models. The impact of motivated physicians and teachers as positive role models on the career choice are recognized in the literature [6, 9]. It is known that prolonged, practical experiences increase both students’ interest in a specialty and the professional fulfillment of teachers [10, 11]. This is also true for optional course work [12]. Accordingly, based on the literature student-clinician mentoring programs might be beneficial for both sides.

Non specialty specific factors, e.g. work-life-balance, workplace conditions and part-time work options, were of high priority to students in our study. This finding is consistent with the literature [4–6]. In contrast, study participants reported poor work-life balance, poor workplace conditions and limited options to work part time. Considering this, one might hypothesize university residency programs inadvertently select against candidates who prioritize work-life balance and in turn raise the concern that high-quality trainees may be lost to more flexible specialties. Underlining this, only 20 % of our cohort work part-time, whereas the average is at 36 % according to a national survey [8]. University hospitals, with complex rotation requirements and research expectations, may be particularly resistant to part-time training models. Given that 54 % of German residents desire part-time work according to a nation wide survey [2] (likely also due to the already high workload) and the repeated demand for part-time options in our study, developing innovative approaches to flexible training represents a strategic imperative rather than an optional enhancement. International examples like the NHS’s “Less Than Full Time training program” demonstrate, despite persisting difficulties, a large scale feasibility in principal [13]. While not directly derived from our data, the literature supports that gender aspects are an additional aspect to consider. Women report especially high concerns for work-life balance, stress and family compatibility [6]. Increasing both options of part-time work and family compatibility may be crucial advantages for a specialty, as approximately two-thirds of medical students are female [14].

In summary, early career interventions should focus on creating positive, high-quality clinical and training experiences, fostering role models and improving workplace conditions and part-time options. However, it is necessary to value and support teaching and mentoring physicians. Dissatisfied, overworked doctors are deterring as several participants have mentioned.

### Enthusiasm and frustration: contrasting perspectives across career stages

#### Current residency and workplace conditions

In our survey medical students were enthusiastic to become doctors and residents were highly committed to their jobs in Neurology. However, they reported poor workplace and residency program conditions. On the basis of our data, many of the needs of the participating residents, e.g. regular feedback, structured mentoring, an open communication culture, appreciation and perceived fairness, are not met. Education is viewed as not satisfactory. The digital infrastructure is inadequate and work overload is the norm. This is also true in nation-wide studies [2, 8]. A model to interpret these reported circumstances is the Job-Demands-Resources Model, which is commonly used to describe job satisfaction. It proposes that high job demands and a lack of job resources lead to exhaustion, disengagement and burnout [15]. Considering the reported workplace and residency conditions in our and nationwide studies, it seems intuitive, that high rates of burnout among neurological residents are reported in the literature [16] and the participating residents in our study expressed dissatisfaction and frustration. Some distress is likely mitigated by the reported good team spirit and team support among residents, which are acknowledge in the literature as a strong job resource [15, 17, 18]. Continuing this pathway will likely be unsustainable. Already 37 % of German residents consider changing professions due to the insufficient workplace conditions [2]. Several actions can be taken to improve these conditions.

#### Improving workplace conditions

Our survey and other studies have identified specific workplace deficits that need improvement [8]. A central issue is the perceived high workload, which in turn is associated with burnout and mental health conditions [19–21]. The high workload is partly due to the heavy clerical burden, as physicians spend three hours per day on documentation [8, 22]. “Traditional” approaches to lower the burden are medical scribe programs, which can increase efficacy as well as physician and patient satisfaction [23]. Based on existing literature a more contemporary and promising approach is the implementation of AI-tools, and Large Language Models (LLMs) in particular, to aid in documentation and to summarize patient-doctor interactions [24, 25]. Such LLMs may aid in improving patient care and differential diagnostics as shown in a recent study [26]. Considering the particularly poor ratings for digital infrastructure in our survey, major investments in digital infrastructure appear to be a prerequisite for further improvements.

Another addressable area is the current leadership and communication culture. The literature recognizes that inadequate leadership and work climate are hazardous to patient safety [27]. It is also associated with physician burnout and dissatisfaction [28]. A positive leadership culture is the “just culture”, characterized by system thinking (focusing on systemic causes of errors not just individual blame), providing psychological safety (staff feels safe to report critical incidents) and promoting learning from errors [29]. Creating an psychologically safe environment fosters learning and performance. Easy steps for leaders in this direction are acknowledging fallibility, asking questions and appreciating other’s contribution [30]. Other helpful and teachable skills of effective leadership are providing feedback, coaching and recognizing good work [28]. Regular feedback meetings and mentoring programs are additional, easily implementable steps to create a open feedback and communication culture and improve feedback quality [31]. Previous examples have been successful in improving physician core competencies and wellness [32, 33]. Considering the reported survey data, the ratings for error culture, feedback culture and the reported negative judgment suggest that elements of a blame culture may be present in our cohort. However, the surveyed residents appear to demand a shift in culture similar to a just culture. Finally, the further implementation of part-time work options and better work-life balance may have positive influences on gaining and retaining employees as described above.

In summary, improving the workplace and digital infrastructure to reduce workload, increase part-time options and improve the leadership and communication culture are points of focus.

#### Improving residency conditions

Less than half of residents in our study are content with the quality of their residency. This is a drop of approximately 20 % compared to a German-wide study from 2018 [34]. Our findings suggest two central issues. The first is an unmet demand for structured and continuous education and feedback. Both can improve the professional development and well-being of residents. While a lack of feedback has been associated with burnout [35], a good and continuous education is to be considered a job resource in the Job-Demands-Resources Model. Empirical data show that learning is negatively associated with burnout and leads to increased satisfaction and engagement [36]. Potential approaches to improve education are simulations, which have proven to be effective [37, 38]. Dedicated protected time in a residency program could facilitate such educational activities. The important role and merits of feedback, especially in a just culture, are listed above.

The second issue in our data is a need for a predictable and fair progress in residency program. Perceived unfairness may be an expression of an underlying closed communication. Implementing a “common trunk” with fixed rotations, as known from surgical fields, might be a solution to improve predictability. This has been proposed previously [34] and may also be helpful for long-term personal planning.

### Limitations and strengths

This study has several limitations. Our sample is not representative of the German healthcare system and is not generalizable to other healthcare systems. Focusing on neurological residents from university hospitals might have introduced bias due to a especially competitive and challenging workplace environment. Additionally, voluntary survey participation may have attracted individuals with particularly strong opinions — either highly satisfied or highly dissatisfied trainees. However, most findings are consistent with other studies on the German or other healthcare systems [2, 6, 8, 20]. We therefore consider our findings to be sound and relevant. While our two cohort approach provides insights into different career stages, we did not track individual career trajectories. This clearly limits our claims. Furthermore, comparing neurological residents with undifferentiated medical students, who have not yet committed to a specialty, limits the interpretability of inter-cohort comparisons, as students do not represent future neurologists but rather a general medical student population. The small sample size does not allow the creation of subgroups for further analysis limiting more nuanced insights. The lack of an external validation of the designed questionnaire may reduce the items representativeness, clarity, and relevance [39]. No formal psychometric testing, including assessment of reliability or construct validity, was performed. This was partially controlled by basing the questionnaire on similar studies and through iterative development by an expert panel composed of clinicians, medical educators and resident representatives. By designing cohort specific questionnaires we ensured a high relevance to the individual context of the cohort but impeded direct quantitative comparisons between cohorts. Lastly, as the analyzing authors are neurologists and medical educators within the German university hospital system they may be subject to bias in the categorization and interpretation of qualitative responses.

## Conclusion

This two cohort study assesses attracting and deterring factors in Neurology training. It reveals that high enthusiasm and commitment among the study participants is met with structural workplace deficits. This is associated with reported frustration and dissatisfaction. Based on literature and key reported issues, intervention targets to address this include digital infrastructure improvement, psychological safety, open feedback and just culture systems, flexible part-time training options, and modern leadership development on a more systemic level. When considering medical education in an narrower sense, developing high-quality clinical teaching experiences including simulations and promoting positive role models are concrete targets. The strong intrinsic motivation observed among residents in our study provides a foundation for retention if training conditions can be aligned with contemporary physician expectations. Evidence-based workplace improvements and systemic reforms could strengthen Neurology’s future workforce. While these interventions are very likely to sustain and boost interest in the specialty, it remains unclear whether these measures are sufficient to secure a sustainable workforce in the face of the broader upheaval currently taking place in medicine.

## Supplementary Information

Supplementary Material 1. Questionnaire for medical students: *Here should be file studierende-umfrage-neuro.pdf*. Questionnaire for neurological residents: *Here should be file umfrage-assistenzaerzt-innen-neurologie.pdf*.

## Data Availability

The data used and analyzed during the current study are available from the corresponding author on reasonable request. Restrictions may apply for data that may compromise anonymity of participants

## Abbreviations

LLM: Large Language Model
TUM: Technical University Munich
