# Supplementary material for: Enthusiasm and structural frustration? Insights from an exploratory cross-sectional two-cohort study into career decision-making and retention factors in German neurology
Source: BMC Med Educ. 2026 Oct 5;26:1586. doi: 10.1186/s12909-026-10515-0 (PMC13640364; doi:10.1186/s12909-026-10515-0)
Supplement: Supplementary file 1 [file 12909_2026_10515_MOESM1_ESM.zip › translation-survey-among-medical-students.pdf]

Translation: Survey among Medical Students

1 What's your gender?

○ female ○ male ○ divers

2 How old are you?

Fragesupport: *Insert only digits*

3 I am a parent

Fragesupport: *Wählen Sie eine Antwort*

○ yes ○ no

4 I would study medicine again

Fragesupport: *Wählen Sie eine Antwort*

○ yes ○ no ○ maybe

5 I am looking forward to practice medicine

○ yes ○ no ○ maybe / do not know ○ I want to do something else anyway (not work as a doctor)

6 I already know which field I want to specialize in after completing my studies...

Fragesupport: *Wählen Sie eine Antwort*

○ yes ○ no ○ maybe/ I have an vague idea

7 I already have an idea of where I want to work in the LONG TERM.

Fragesupport: *Select the answer that best matches your current opinion!*

- ○ practice/medical care center self-employed
- ○ practice/medical care center employed
- ○ Non-university hospital
- ○ University hospital
- ○ no idea
- ○ other [ ]

8 The following points, experiences, or people will (probably) play a role in my decision regarding my future career:

Fragesupport: *1=not at all 5=very strongly*

	1	2	3	4	5
Role Models among Physicians	○	○	○	○	○
Role Models among Lecturers	○	○	○	○	○
Influence by family/ fruends	○	○	○	○	○
good teaching	○	○	○	○	○
Working conditions in general	○	○	○	○	○
regular working hours	○	○	○	○	○
option to work part time	○	○	○	○	○
Compatibility with private life	○	○	○	○	○
structured specialist medical training	○	○	○	○	○
meaningful/regular feedback	○	○	○	○	○
error culture	○	○	○	○	○
digitization	○	○	○	○	○
Specialty itself	○	○	○	○	○
PhD-Thesis	○	○	○	○	○

student assistant job	○	○	○	○	○
participation in congress	○	○	○	○	○
Place of wokr	○	○	○	○	○
additional courses in clinic/ university	○	○	○	○	○
Contact or collaboration with professional associations (e.g., Young Neurologists) or similar organizations	○	○	○	○	○

9 This is what particularly inspired me during my education for a subject/clinic/institute.

10 This is what particularly put me off a particular subject/clinic/institute:

11 This is also important to me regarding the topic/other comments
