# Supplementary material for: Enthusiasm and structural frustration? Insights from an exploratory cross-sectional two-cohort study into career decision-making and retention factors in German neurology
Source: BMC Med Educ. 2026 Oct 5;26:1586. doi: 10.1186/s12909-026-10515-0 (PMC13640364; doi:10.1186/s12909-026-10515-0)
Supplement: Supplementary file 1 [file 12909_2026_10515_MOESM1_ESM.zip › translation-survey-among-neurological-residents.pdf]

Translation: Survey among Neurological Residents

1 What is your gender?

Fragesupport: *Wählen Sie eine Antwort*

○ female ○ male ○ divers

2 How old are you?

Fragesupport: *Only insert digits*

[ ]

3 Are you a parent?

Fragesupport: *Wählen Sie eine Antwort*

○ Yes ○ No

4 I have been a neurological resident in my

Fragesupport: *Wählen Sie eine Antwort*

○ 1.-2. year ○ 3.+4. year ○ 5.+6. year ○ 7. year or more ○ I'm already a specialist

5 I work

Fragesupport: *Wählen Sie eine oder mehr Antworten*

☐ fulltime ☐ part time

☐ part time with the following hours [ ]

6 After completing my residency, my long-term goal is to work

Fragesupport: *Please choose what you deem most likely*

- ○ in a practice/medical care center in employment
- ○ in a practice/medical care center in self-employment
- ○ as an attending/ senior physician at a university hospital
- ○ chief physician at university hospital
- ○ attending/ senior physician at non-universitiy hospital
- ○ chief physician at non-universitiy hospital
- ○ purely scientifically
- ○ I do not know
- ○ Sonstiges, nämlich: [ ]

7 My specialist training ... (for specialists, please answer retrospectively)...

Fragesupport: *1 = not at all, 5 = very satisfactory*

	1	2	3	4	5
is satisfactory in terms of quality	○	○	○	○	○
is satisfactory in terms of timing	○	○	○	○	○
proceeds according to a clear plan that appears fair	○	○	○	○	○

8 Specialist training at our institution generally/usually takes the following number of years:

Fragesupport: *Only insert digits.*

[ ]

9 What do you consider to be a good specialist training?

Fragesupport: *e.g., strict rotation schedule, teaching rounds, continuing education...*

[ ]

10 I am supported in my professional training and development.

Fragesupport: *1=not at all 5=great support*

☆☆☆☆☆ [ ] / 5

11 I enjoy my work

Fragesupport: *1= not at all 5= very much*

☆☆☆☆☆ [ ] / 5

12 I am prepared to go above and beyond what is required.

Fragesupport: *1=not at all 5= very much*

☆☆☆☆☆ [ ] / 5

13 We provide good patient care

Fragesupport: *1=not at all 5=great*

☆☆☆☆☆ [ ] / 5

14 I feel that my colleagues are a good team/we work with good team spirit.

Fragesupport: *1=not at all 5=very much*

	1	2	3	4	5
in general	○	○	○	○	○
with other residents	○	○	○	○	○
with our attendings	○	○	○	○	○
with nursing and other professional groups	○	○	○	○	○

15 I receive appreciation for my work.

Fragesupport: *1=not at all 5= very much*

☆☆☆☆☆ [ ] / 5

16 I receive helpful feedback on my work (on a regular basis)

Fragesupport: *1=not at all 5= very much*

☆☆☆☆☆ [ ] / 5

17 I would like more professional or personal feedback on my work.

Fragesupport: *1=not at all 5= very much*

☆☆☆☆☆ [ ] / 5

18 I can communicate my problems, needs, or medical questions without having to expect negative judgment.

Fragesupport: *1=not at all 5= very much*

☆☆☆☆☆ [ ] / 5

19 There's an sufficient error culture

Fragesupport: *1=not at all 5= very good*

☆☆☆☆☆ [ ] / 5

20 I have confidence in my superiors and management, and I can understand developments and decisions if I want to.

Fragesupport: *1=not at all 5= very much*

☆☆☆☆☆ [ ] / 5

21 Physicians are constantly overworked at our department

Fragesupport: *1=not at all 5= constantly*

☆☆☆☆☆ [ ] / 5

22 I can balance my work and personal life well.

Fragesupport: *1=not at all 5= very well*

☆☆☆☆☆ [ ] / 5

23 There are meaningful part-time opportunities/part-time offers.

Fragesupport: *Select one or more answers*

☐ yes, but only for some ☐ yes, for all ☐ no

☐ if yes, which part-time offers? [ ]

24 I find my work environment to be good and helpful in terms of digitalization/IT.

Fragesupport: *1=not at all 5= very much*

☆☆☆☆☆ [ ] / 5

25 I find my work environment helpful in terms of the division of labor with other professional groups (e.g., nursing staff, ward assistants, PAs, etc.).

Fragesupport: *1=not at all 5= very much*

☆☆☆☆☆ [ ] / 5

26 Overtime/extra hours are compensated...

Fragesupport: *Choose one or more answers*

☐ no ☐ in part (e.g. max. hours per month) ☐ yes, as performed ☐ exclusively with time off in lieu

☐ solely financial ☐ with time off in lieu and/or financially, can be chosen ☐ with time off in lieu and/or financially, as specified by the employer ☐ Don't know how overtime is compensated

☐ other [ ]

27 I have the feeling that...

Fragesupport: *Wählen Sie eine oder mehr Antworten*

☐ Something is being done to improve employee satisfaction. ☐ Something is being done to promote medium- to long-term employee retention, and reflection is taking place on this.

☐ What is being done to promote satisfaction and employee retention? [ ]

28 My hospital/ department makes a positive contribution to recruiting young talent in the medical field (e.g., information days, advertising for practical training years, good teaching, good clinical internships, etc.).

Fragesupport: *1=not at all 5= very much*

☆☆☆☆☆ [ ] / 5

29 If something is being done to recruit young talent, what is being done?

[ ]

30 How important are the following areas for a GOOD workplace for you?

Fragesupport: *1=not important 5= very important*

	1	2	3	4	5
good continuous education	○	○	○	○	○
Digitization/ IT	○	○	○	○	○
Division of Labor	○	○	○	○	○
work life ballacne	○	○	○	○	○
Option to work part time	○	○	○	○	○
regular working hours	○	○	○	○	○
few shifts (weekend, night)	○	○	○	○	○
Career opportunities/promotion prospects	○	○	○	○	○
Career planning mentoring	○	○	○	○	○

31 The most important factors in deciding to pursue neurology were...

Fragesupport: *1=not important at all 5=very important*

	1	2	3	4	5
Specialty itself	○	○	○	○	○
good teaching	○	○	○	○	○
role models among lecturers	○	○	○	○	○
role models among physicians	○	○	○	○	○
Physicians	○	○	○	○	○
other reasons	○	○	○	○	○

32 I would choose Neurology as a specialty again

Fragesupport: *Wählen Sie eine Antwort*

○ yes ○ no ○ maybe

33 I would choose my current employer again

Fragesupport: *Wählen Sie eine Antwort*

○ yes ○ no ○ maybe

34 What should be improved/changed at my workplace in the near future?

35 What should be the first thing to change/improve in health policy?

36 What else is important to you on this topic? Or is there anything else you would like to emphasize?
